# Solidification of Lithium Slag via Microbial-Induced Carbonate Precipitation Mediated by Urease-Producing Bacteria

**DOI:** 10.3390/microorganisms14102194

**Published:** 2026-10-01

**Authors:** Wei Dong, Luyao Wang, Shaoqing Lei, Yuexin Song, Qian Zhou

**Affiliations:** 1 Yichun Lithium New Energy Industry Research Institute, Jiangxi University of Science and Technology, Yichun 336023, China; wdong@jxust.edu.cn; 2 School of Mining Engineering, Jiangxi University of Science and Technology, Ganzhou 341000, China; 6120240260@mail.jxust.edu.cn (L.W.); 1120220437@mail.jxust.edu.cn (S.L.); 6120220413@mail.jxust.edu.cn (Y.S.); 3 Jiangxi Provincial Key Laboratory of Environmental Pollution Prevention and Control in Mining and Metallurgy, Ganzhou 341000, China; 4 School of Environmental Engineering, Jiangxi University of Science and Technology, Ganzhou 341000, China

**Keywords:** urease-producing bacteria, lithium slag, biocementation, crystalline calcite, stabilization

## Abstract

The rapid development of the new energy industry has led to a sharp increase in the demand for lithium resources, resulting in a substantial accumulation of lithium smelting residue (lithium slag). Conventional disposal methods like landfilling and open-air storage may cause potential environmental concerns, including the migration of soluble components and long-term ecological risks. Microbial-induced carbonate precipitation (MICP) presents an alternative biologically based stabilization strategy for waste stabilization by leveraging biomineralization to convert reactive components into stable precipitates. Herein, the feasibility of solidifying lithium slag (LS) via MICP was investigated using urease-producing bacteria with contrasting enzyme activities, including a laboratory-isolated strain *Lysinibacillus* sp. WD018 and a commercial strain *Sporosarcina pasteurii* (*S. pasteurii*). Due to its superior urease activity (45.25 vs. 2.25 mM/min/OD_600_), *S. pasteurii* induced markedly greater carbonate precipitation, resulting in higher CaCO_3_ production and the formation of more crystalline calcite. Among the tested conditions, a cylindrical flow-through mold, 1 M cementation solution, and seven treatment cycles were favorable for producing intact LS specimens. X-ray diffraction (XRD) indicated calcite formation after MICP treatment, and *S. pasteurii* yielded more crystalline calcite than WD018. The CaCO_3_ content was higher in the upper section (51 ± 0.91% for *S. pasteurii* vs. 32 ± 0.10% for WD018). Untreated LS had permeability coefficients of (1.71 × 10^−6^)~(1.13 × 10^−5^) m/s, whereas treated specimens showed no visible water seepage during a 30 min constant-head test. These findings indicated that bacterial urease activity and flow-through treatment configuration are important factors controlling carbonate precipitation and LS stabilization.

## 1. Introduction

The global transition to green and low-carbon economies has established lithium as a critical raw material for lithium-ion batteries, thereby driving the rapid expansion of industries such as new energy vehicles and sustaining worldwide demand for the metal [1,2]. Meeting this growing demand relies predominantly on the extraction of lithium from hard-rock minerals, which produces substantial quantities of solid waste. Notably, the production of 1 ton of lithium salt generates approximately 8–10 tons of lithium slag (LS). In China alone, millions of tons of LS accumulate annually, with total stockpiles reaching tens of millions of tons [3,4]. Currently, LS is predominantly disposed of in landfills or stored in open air, practices that not only consume valuable land resources but also pose severe environmental risks. Rainwater leaching can mobilize heavy metals and other pollutants from the slag into soils and groundwater, threatening ecosystems and human health [5,6].

LS is recovered mainly by utilizing it as a mineral admixture in cement and concrete, providing a large-scale, cost-effective, and environmentally friendly solution for waste management [7,8,9]. It can serve as a supplementary cementitious material in concrete and mortar, partially replacing cement. Through its hydration reactions and physical filler effects, LS can enhance the mechanical properties (e.g., compressive strength), compactness, and durability [10,11,12]. However, effectively utilizing LS typically requires activation. While physical activation methods (e.g., grinding) are mature and straightforward, they are often energy-intensive. Meanwhile, chemical activation methods can enhance mechanical performance and impart beneficial properties such as anti-corrosion and anti-carbonation resistance. Nevertheless, it remains practically challenging because chemical activators may interact with multiple concrete constituents, complicating the accurate determination of their actual activation effects [4]. Therefore, developing a more efficient and environmentally compatible utilization strategy for LS is highly desirable.

Microbial-induced carbonate precipitation (MICP) has emerged as a biologically based stabilization strategy that facilitates the formation of calcium carbonate (CaCO_3_) precipitates through microbial metabolic activities, including urea hydrolysis, sulfate reduction, and denitrification [13]. Compared to traditional physical and chemical methods, MICP offers advantages such as lower energy consumption, environmental compatibility, and operational simplicity [14]. Of the various pathways for MICP, ureolysis-driven MICP, where urease-producing bacteria hydrolyze urea to generate carbonate ions (CO_3_^2−^), is particularly prominent for its high efficiency. It has been widely applied in soil reinforcement and heavy metal remediation, whereas its application to LS solidification remains scarce [15,16,17]. Recently, Li et al. demonstrated that MICP can improve the mechanical strength of LS and immobilize Be and Tl under both room- and low-temperature conditions, confirming the feasibility of microbial solidification for LS treatment [18]. The potential of MICP lies in its capacity to induce CaCO_3_ precipitation, which can bind loose particles and fill internal pores. Since LS contains reactive calcium-bearing phases like gypsum (CaSO_4_·2H_2_O) and lithium pyroxene (LiAlSi_2_O_6_) that can serve as potential calcium sources, MICP presents a viable strategy for the solidification and stabilization of LS [19].

**Scheme 1:**
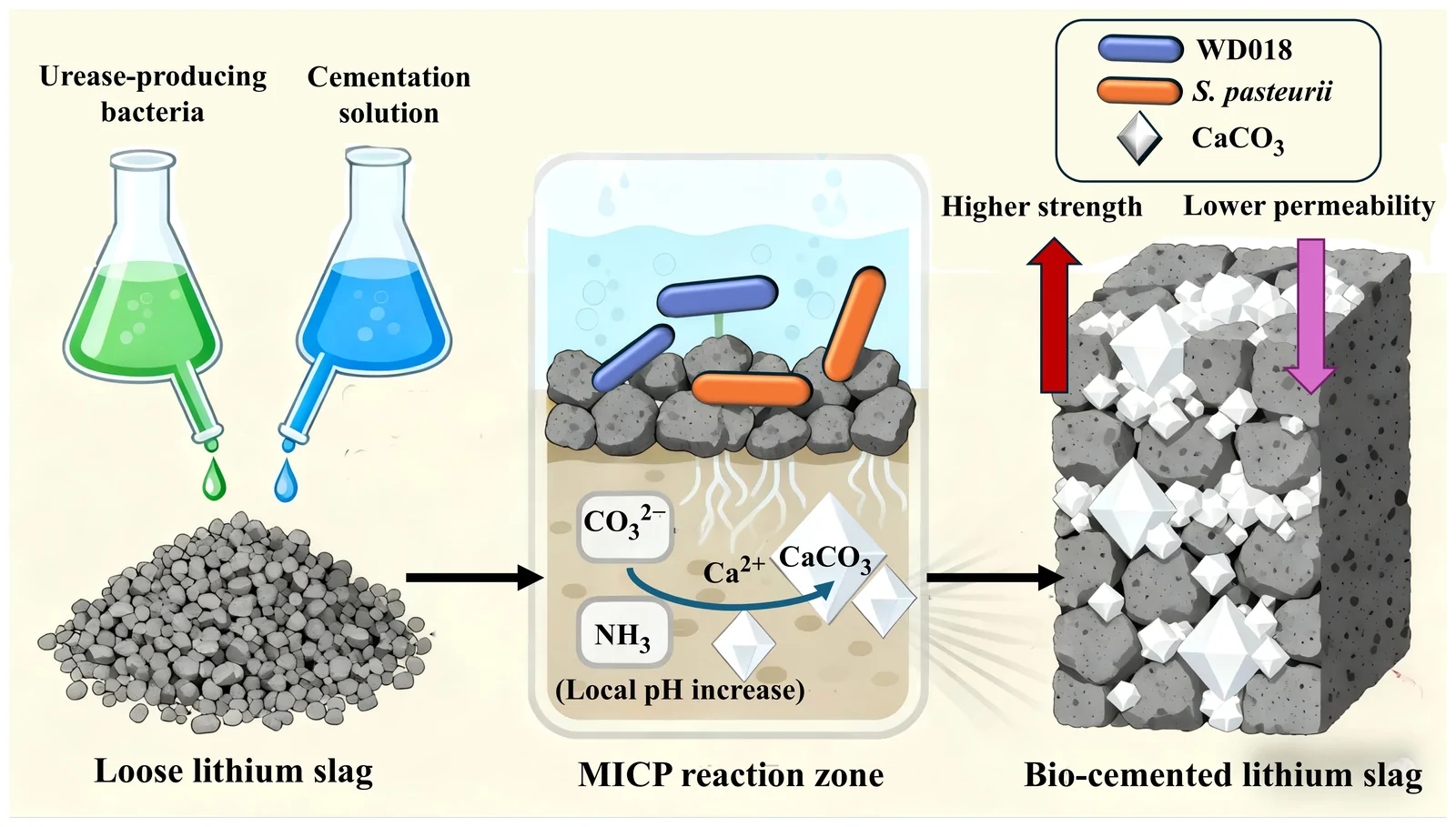
Proposed mechanism of LS solidification via ureolysis-driven MICP. Upward and downward arrows indicate increases and decreases, respectively, in the corresponding performance or process characteristics.

The core mechanism of LS solidification via MICP, as illustrated in Scheme 1, involves three interconnected stages. Initially, urease-producing bacteria are introduced along with a cementation solution containing urea and calcium ions (Ca^2+^). The bacterial urease hydrolyzes urea to produce CO_3_^2−^ and ammonium ions (NH_4_^+^), thereby elevating the local pH. Subsequently, the generated CO_3_^2−^ reacts with available Ca^2+^ (sourced from the cementation solution and/or dissolved from calcium-bearing phases in the slag) to form CaCO_3_ precipitates, which are favored and stabilized under alkaline conditions. Ultimately, the precipitated CaCO_3_ crystals fill the pores in the LS matrix and promote particle bonding, thereby improving the consolidation performance and reducing water permeability of the treated matrix. MICP offers advantages over conventional physical and chemical methods, including lower energy consumption, environmental compatibility, and operational simplicity. Both selected strains are spore-forming, urease-producing bacteria with strong stress tolerance, which may favor survival and mineralization in solid waste matrices. However, the role of intrinsic bacterial urease activity in controlling the extent, crystallinity, and spatial uniformity of carbonate precipitation in LS remains poorly understood, and the relative contributions of treatment configuration (non-flow-through vs. flow-through), cementation solution concentration, and number of treatment cycles have not been examined together. Previous studies have addressed some of these factors individually, but direct comparisons between laboratory-isolated ureolytic strains and *Sporosarcina pasteurii* (*S. pasteurii*) under comparable LS solidification conditions remain limited. To address these gaps, we first investigated bacterial growth, urease activity, and spore preparation in the two strains. We then evaluated LS solidification under non-flow-through and flow-through configurations and examined the effects of cementation solution concentration and treatment cycles. Finally, we related the observed solidification performance to CaCO_3_ precipitation, microstructure, and mineral phase changes.

## 2. Materials and Methods

### 2.1. Bacterial Strains and Cultivation

Two urease-producing bacterial strains were used: a urease-producing strain isolated from ionic rare-earth tailings in Ganzhou, China, and subsequently designated as *Lysinibacillus* sp. WD018, and the commercial *S. pasteurii* (ATCC 11859) purchased from the Beijing Microbiology Culture Collection Center (Beijing, China) [20]. WD018 was activated on Luria–Bertani (LB) solid medium (containing tryptone (Beijing Aoboxing Biotechnology Co., Ltd., Beijing, China) 10 g/L, yeast extract (Beijing Aoboxing Biotechnology Co., Ltd., Beijing, China) 5 g/L, and NaCl (Xilong Scientific Co., Ltd., Shantou, China) 10 g/L) [21] and then cultured in LB liquid medium at 37 °C with shaking at 180 rpm. *S. pasteurii* was activated and cultured in Casein Soybean Digest (CASO) medium (containing casein peptone (Beijing Aoboxing Biotechnology Co., Ltd., Beijing, China) 15 g/L, soymeal peptone (Beijing Aoboxing Biotechnology Co., Ltd., Beijing, China) 5 g/L, and NaCl 5 g/L). After autoclaving and cooling to about 50 °C, 100 mL of filter-sterilized 20% (*w*/*v*) urea (Xilong Chemical Co., Ltd., Shantou, China) solution was added to give a final urea concentration of 2% at 30 °C, 180 rpm. Both strains were grown to the logarithmic growth phase for subsequent experiments.

### 2.2. LS Preparation

Lepidolite-based LS was obtained from a lithium smelter in Yichun, Jiangxi Province, China. Previous studies have shown that lepidolite-derived LS is mainly composed of SiO_2_, Al_2_O_3_, CaO, and SO_3_, together with minor amounts of alkali and alkaline-earth metal oxides. In addition, trace amounts of potentially hazardous elements (e.g., Cr, Ni, Cu, Zn, As, Cd, Hg, and Pb) may remain in the slag depending on the lithium extraction process, which has raised increasing concerns regarding its environmental management and safe utilization [22]. Therefore, effective stabilization of LS is of considerable environmental significance. The raw slag was ground to pass through a 0.7 mm sieve and dried at 80 °C for 24 h before use.

### 2.3. Growth Curves and Urease Activity Determination

Bacterial growth was monitored by measuring the optical density at 600 nm (OD_600_) hourly using a microplate reader (SuPerMax300, Shanghai Flash Spectrum Biotechnology Co., Ltd., Shanghai, China). All growth and urease activity experiments were performed with three independent replicates (*n* = 3). Urease activity was determined by the conductivity method as described previously [23]. Briefly, 1 mL of bacterial culture was mixed with 9 mL of 1.5 M urea solution. The change in conductivity was recorded at 25 °C for 5 min. Urease activity (U, mM/min/OD_600_) was calculated and standardized to pH 7.0 using established formulas [20].

### 2.4. Spore Preparation and Germination Assay

Pure spores were prepared by inoculating logarithmic-phase cultures into a specialized Spore Induction-Calcium Glucose Nutrient Medium (SG medium contained nutrient broth (Sinopharm Chemical Reagent Co., Ltd., Shanghai, China) 16 g/L, KCl (Sinopharm Chemical Reagent Co., Ltd., Shanghai, China) 2.0 g/L, MgSO_4_·7H_2_O (Sinopharm Chemical Reagent Co., Ltd., Shanghai, China) 0.5 g/L, Ca(NO_3_)_2_ (Sinopharm Chemical Reagent Co., Ltd., Shanghai, China) 1 mM, MnCl_2_ (Sinopharm Chemical Reagent Co., Ltd., Shanghai, China) 0.1 mM, FeSO_4_ (Sinopharm Chemical Reagent Co., Ltd., Shanghai, China) 1 μM, and glucose (Sinopharm Chemical Reagent Co., Ltd., Shanghai, China) 1 g/L) and incubating at 30 °C, 180 rpm for 24 h [24]. The spores were purified via repeated washing and ultrasonication to remove vegetative cells, resulting in a spore suspension with purity higher than 98%.

For germination tests, the spore suspension (OD_600_ = 0.6–1.0) was mixed with 10 mM HEPES buffer (Shanghai Aladdin Biochemical Technology Co., Ltd., Shanghai, China) (pH 7.4) supplemented with glycine (Sinopharm Chemical Reagent Co., Ltd., Shanghai, China), alanine (Sinopharm Chemical Reagent Co., Ltd., Shanghai, China), or valine (Sinopharm Chemical Reagent Co., Ltd., Shanghai, China), each at 10 mM. Germination was assessed by monitoring OD_600_ changes and observing spore darkening under phase-contrast microscopy (Nikon Instruments (Shanghai) Co., Ltd., Shanghai, China). Each germination condition was tested with three independent replicates (*n* = 3).

### 2.5. Solidification Experiment and Mold Design

Solidification efficacy was evaluated using two treatment configurations: a non-flow-through system and a flow-through system. A square silicone mold (20 mm × 20 mm) with a closed bottom was used as the non-flow-through system for small-scale tests with 6 g of LS. A cylindrical polyvinyl chloride (PVC) mold (40 mm diameter) with a filter membrane at the bottom was used as the flow-through system for larger-scale tests with 46 g of LS. In the flow-through system, solutions were percolated from top to bottom using a peristaltic pump at a flow rate of 400 μL/min. The two systems differed not only in geometry but also in specimen mass, drainage, and flow conditions. Therefore, they were compared as non-flow-through vs. flow-through treatment configurations rather than as mold geometry alone. The square mold was intended to approximate a relatively static condition with limited external solution exchange, whereas the cylindrical PVC mold was designed to represent a more permeable engineering scenario in which bacterial suspension and cementation solution could penetrate the specimen more uniformly. This dual-configuration approach enabled assessment of how mass-transfer conditions influence bacterial activity, calcium carbonate (CaCO_3_) precipitation, and the spatial distribution of mineralization within the LS matrix.

Three cementation solution concentrations (1, 1.5, and 2 M) and two treatment-cycle numbers (three and seven) were selected. The effects of cementation solution concentration and treatment cycles on CaCO_3_ yield, surface clogging, and the spatial uniformity of cementation were then evaluated under these conditions. These levels were informed by preliminary experiments, which produced clearly distinguishable solidification outcomes while remaining within practically deliverable ranges for the flow-through setup. For each treatment cycle, 20 mL of bacterial suspension was first injected into the specimen and retained for 6 h. Then, 20 mL of cementation solution was injected. The treatment was repeated for three or seven cycles at 24 h intervals. All samples were cured for 21 d and then dried at 80 °C for 24 h before demolding. Each solidification condition was prepared in triplicate (*n* = 3), and representative specimens were presented in the figures.

### 2.6. Characterization of Solidified Products

Microstructural analysis: The microstructure, distribution of CaCO_3_ precipitates, and pore-filling characteristics of the demolded samples were examined using a Philips XL30 W/TMP scanning electron microscope (SEM) (Philips, Eindhoven, the Netherlands). Small pieces of the samples were sputter-coated with a gold layer prior to observation to enhance conductivity and imaging quality.

Phase composition analysis: The mineral phase composition of the solidified products was identified by X-ray diffraction (XRD) using a Bruker D8 Advance diffractometer (Bruker, Karlsruhe, Germany). The samples were ground into fine powder and scanned over a 2θ range of 10–90°. Phase identification was accomplished by comparing the obtained diffraction patterns with standard reference data from the PDF database using Jade 6.0 software. All XRD measurements were performed under identical instrumental conditions, including the same scanning range, step size, scan rate, tube voltage, and tube current. Therefore, peak intensities were compared qualitatively without additional normalization.

CaCO_3_ content: The CaCO_3_ content was determined according to the method described in reference [25].

Permeability coefficient: The permeability coefficient was calculated following the constant-head permeability test method outlined in a previous study based on Darcy’s law [26]. Untreated LS and MICP-treated specimens were placed in a permeameter, and deionized water was supplied under a constant head. The volume of outflow water was recorded over 30 min. The constant-head test was repeated three times for each condition. The permeability coefficient was calculated as *k* = *QL*/(*Aht*), where *Q* is the volume of outflow water, *L* is the specimen height, *A* is the cross-sectional area, *h* is the hydraulic head difference, and *t* is the test duration.

## 3. Results and Discussion

### 3.1. Biological Characteristics and Spore Germination of the Strains

#### 3.1.1. Strain Identification

**Figure 1:**
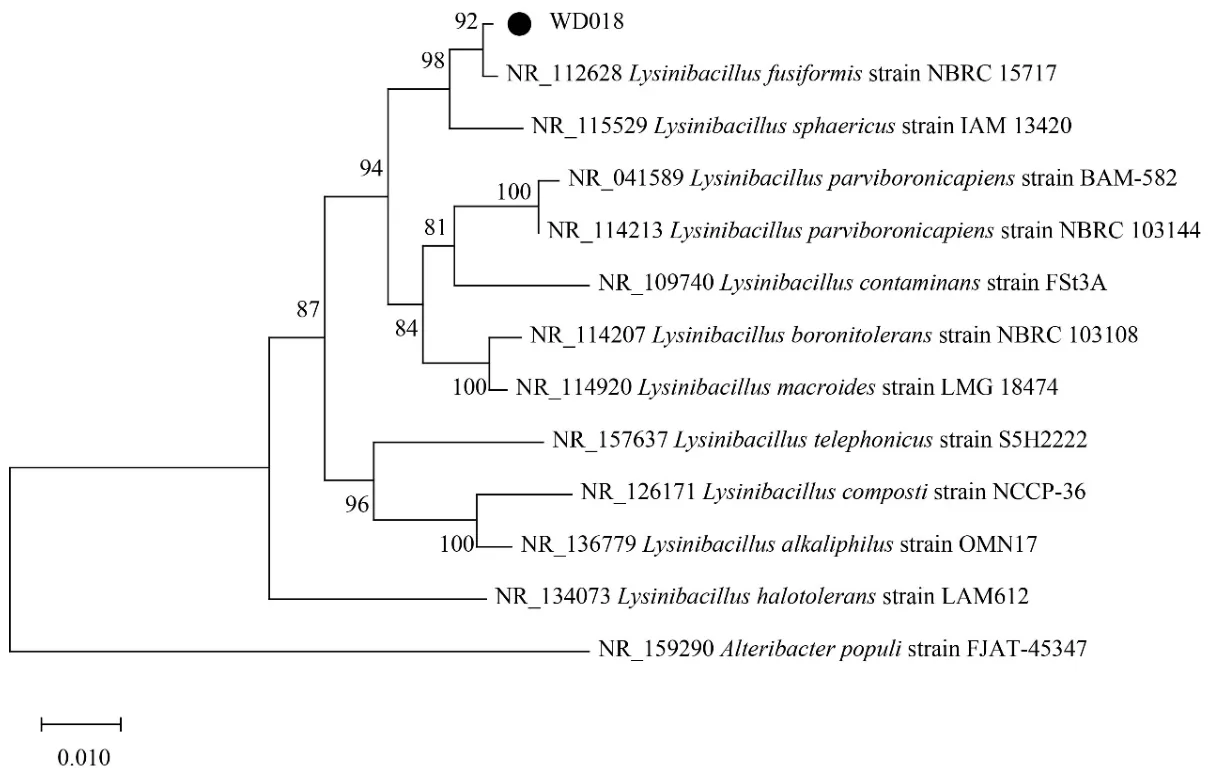
Phylogenetic tree of strain WD018 based on partial 16S rRNA gene sequences. The 16S rRNA sequence of WD018 was compared with those of related *Lysinibacillus* sp. type strains by BLAST against the NCBI 16S rRNA database. The most closely related reference sequences were aligned using MAFFT, and a maximum-likelihood tree was constructed using FastTree. Bootstrap support values are indicated at the nodes, and the black dot indicated strain WD018, *Alteribacter populi* FJAT-45347 (NR_159290) was used as the outgroup. The scale bar represents 0.010 substitutions per nucleotide position.

The laboratory-isolated strain WD018 was identified based on 16S rRNA gene sequencing and phylogenetic analysis. As shown in Figure 1, the partial 16S rRNA gene sequence of WD018 (1350 bp) has been deposited in GenBank under accession number QB025668. A Basic Local Alignment Search Tool (BLAST (version 2.2.28)) comparison against the National Center for Biotechnology Information (NCBI) 16S rRNA database showed that this sequence shared 99.9% similarity (1297 out of 1298 aligned positions, with no gaps) with *L. fusiformis* strain NBRC 15717 (GenBank NR_112628) and 99.8% similarity (1325/1327 positions) with the same type strain deposited as DSM 2898 (GenBank NR_042072); both values are well above the generally accepted 98.65% threshold for species delineation [27]. For phylogenetic reconstruction, the most closely related reference sequences retrieved from NCBI were aligned using Multiple Alignment using Fast Fourier Transform (MAFFT) (version 7.520), and a maximum-likelihood tree was constructed using FastTree (version 2.1.11), with *Alteribacter populi* FJAT-45347 (NR_159290) used as the outgroup. In the resulting tree (Figure 1), WD018 clustered with *L. fusiformis* NBRC 15717 with a bootstrap value of 92, further supporting this species-level assignment. This molecular identification was consistent with its observed colonial morphology (white, circular, and flat colonies) and physiological characteristics (rod-shaped cells). The commercial strain *S. pasteurii* (ATCC 11859) was used as a reference without further identification [20].

#### 3.1.2. Growth Curves and Urease Activity

**Figure 2:**
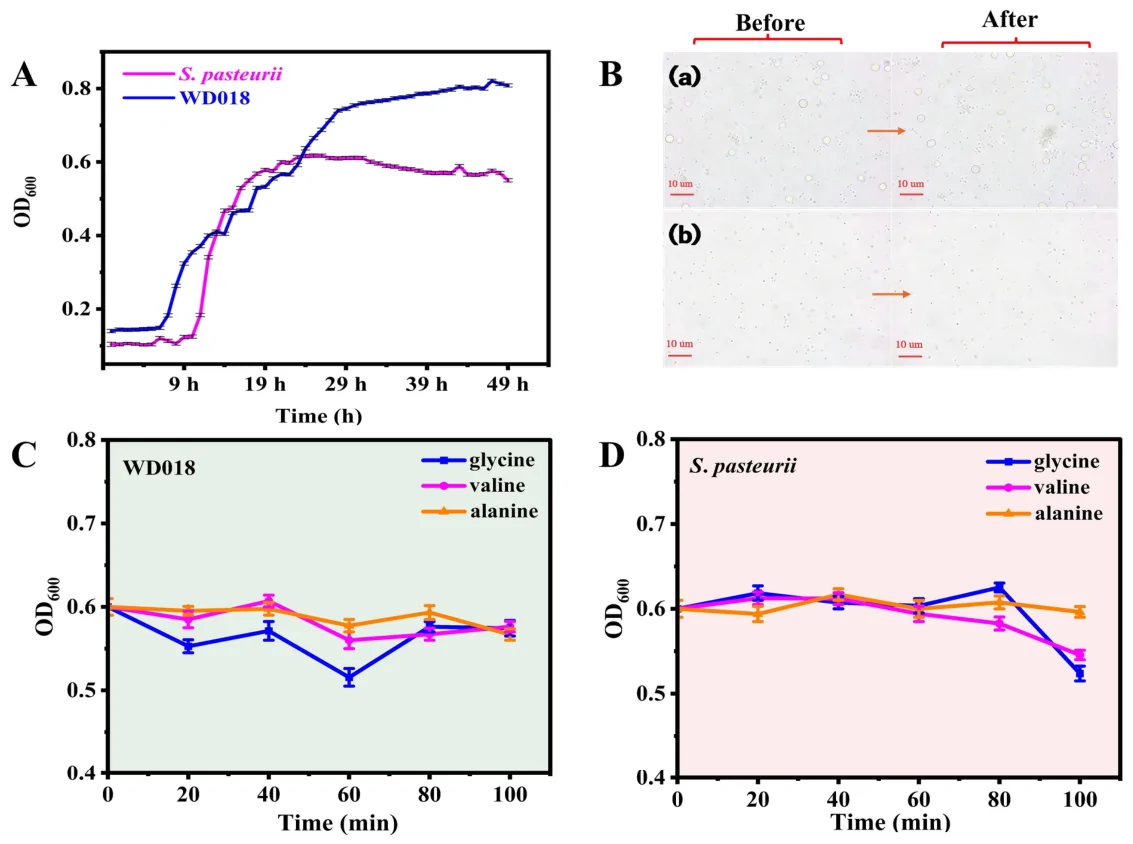
Growth curves and spore germination characteristics of two urease-producing bacteria. (**A**) Growth curves of the two strains; (**B**) Phase-contrast microscopy observation of spore germination, (**a**) WD018 and (**b**) *S. pasteurii*; (**C**) Dynamic OD_600_ change during spore germination of WD018; (**D**) Dynamic OD_600_ change during spore germination of *S. pasteurii*. Error bars in panels (**A**,**C**,**D**) represent the standard deviation of three independent replicates (*n* = 3).

Pronounced interspecific differences were observed in the growth kinetics and urease activity of the two urease-producing strains. The peaks of enzyme activity synchronized with key growth phases, providing a quantitative basis for the application of bacterial suspensions in subsequent solidification experiments. As depicted in Figure 2A, WD018 entered the logarithmic growth phase after 9 h and reached the stationary phase around 30 h at 37 °C with shaking at 180 rpm, with the OD_600_ value maintained between 0.6 and 0.7. *S. pasteurii*, cultured at 30 °C and 180 rpm, exhibited a slightly slower growth rate, entering the logarithmic phase at 12 h and the stationary phase at 20 h. However, it achieved a higher final biomass with an OD_600_ of 0.8–0.9, indicating its robust metabolic potential under favorable culture conditions. Urease activity assays revealed that both strains attained peak enzymatic activity during the late logarithmic phase, specifically between 24–30 h for WD018 and 20–24 h for *S. pasteurii*. A pronounced difference in urease activity was observed: *S. pasteurii* exhibited approximately 20-fold higher urease activity (45.25 mM/min/OD_600_) than WD018 (2.25 mM/min/OD_600_). This superior enzymatic performance holds great promise for solidification.

#### 3.1.3. Spore Preparation and Germination Characteristics

Efficient preparation of pure spores is important for exploring spore-based inoculum strategies in MICP applications, because spores generally exhibit stronger resistance to environmental stresses than vegetative cells [28,29]. Among LB, CASO, and SG media, SG medium exhibited clear advantages for spore preparation. Inoculation of logarithmic-phase cultures into SG medium, followed by incubation at 30 °C and 180 rpm for 24 h, yielded spore suspensions with purity exceeding 98%. In contrast, achieving comparable purity using LB or CASO medium required over 5 d and up to 5 additional washing cycles, indicating that SG medium was more suitable for rapid preparation of high-purity spores of both strains.

Dormant spores are metabolically inactive and do not produce urease. Ureolytic activity is restored only after germination and outgrowth into vegetative cells [29,30]. Efficient germination is therefore essential if spores are to be used as stress-tolerant MICP inocula. Spore germination of the two urease-producing strains was assayed with glycine, alanine, or valine (10 mM each) in 10 mM HEPES buffer (pH 7.4). Phase-contrast microscopy showed that spores of both strains remained phase-bright and spherical throughout the incubation period, with no evident transition to phase-dark spores or emergence of vegetative cells (Figure 2B(a,b)). Consistent with this observation, the OD_600_ values in Figure 2C,D remained stable between 0.6 and 1.0 for 80 min after germinant addition, lacking the characteristic decrease associated with germination-induced hydration and swelling. Collectively, these observations indicate that glycine, alanine, and valine did not appreciably trigger germination of either strain under the tested conditions. Identification of more effective germination triggers is therefore required before these stress-resistant spores can be deployed as inocula, which would lay a foundation for spore-based MICP applications in adverse solid-waste environments.

### 3.2. Evaluation of Solidification Performance Under Different Conditions

#### 3.2.1. Solidification Performance of LS in Molds with Different Flowability

**Figure 3:**
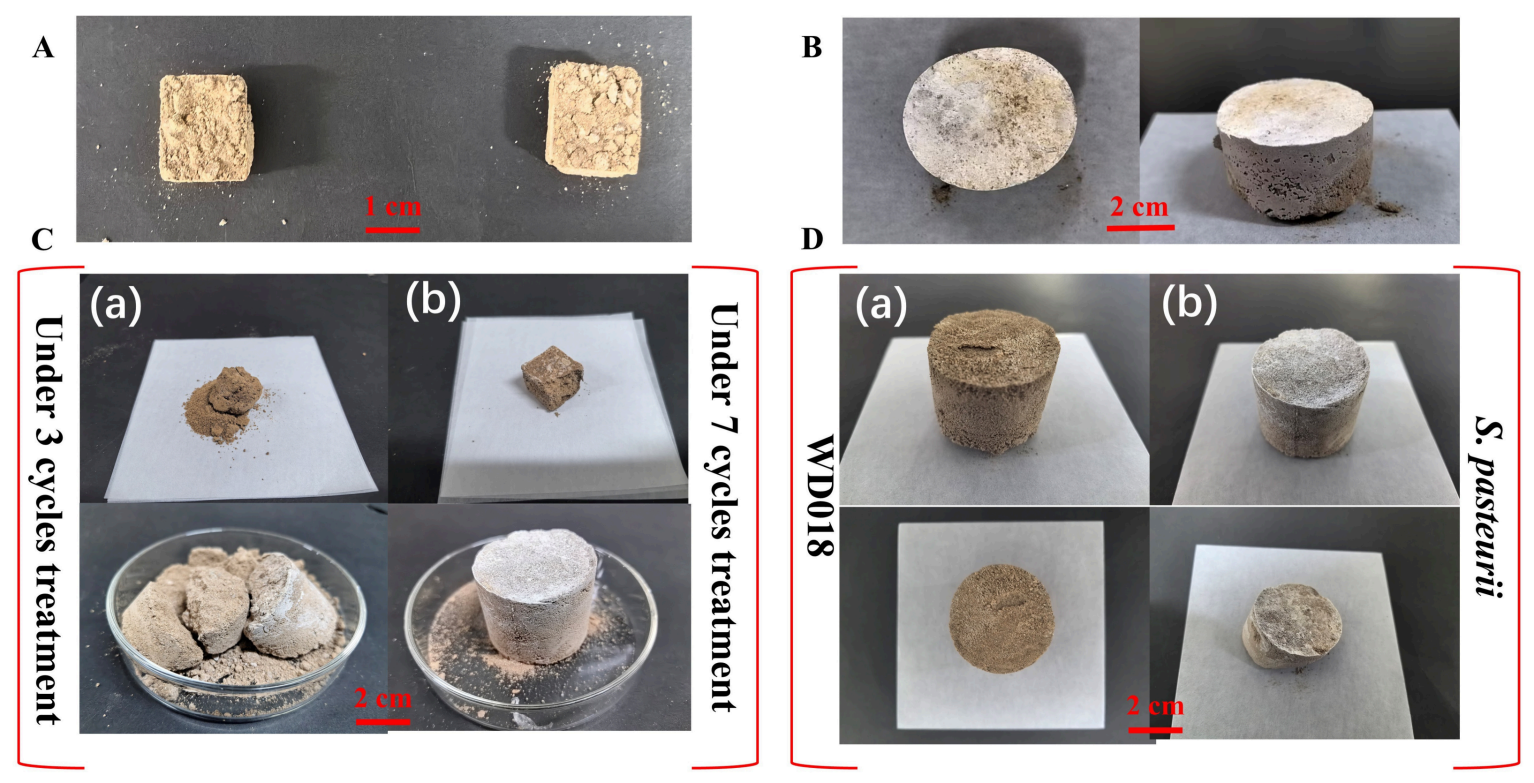
Macroscopic solidification effects of LS under different conditions. (**A**) Solidified product in the square non-flow-through mold; (**B**) Solidified product in the cylindrical flow-through mold; (**C**) Solidified products of *S. pasteurii* under different treatment cycles: (**a**) three treatment cycles and (**b**) seven treatment cycles; (**D**) Comparison of solidified products under seven treatment cycles obtained using different strains: (**a**) WD018 and (**b**) *S. pasteurii*.

Mold design may influence oxygen supply and solution percolation, thereby influencing bacterial metabolic activity and the distribution of CaCO_3_ precipitation [31]. As shown in Figure 3A, in the square silicone mold (a closed-bottom, non-flow-through system), 6 g of LS failed to form a fully solidified monolith after 21 d of curing. Only the top 1–2 mm region formed a slight crust, while the middle and lower layers remained loose. The poor consolidation can be attributed to the lack of drainage in the non-flow-through system, which may have resulted in restricted solution movement and reduced local oxygen availability in the middle and lower sections. Since both bacterial strains are obligate aerobes, restricted oxygen availability may have affected their urease activity and metabolic processes, resulting in the induction of only minimal amounts of loosely aggregated precipitates [32,33].

In contrast, the cylindrical PVC mold (a flow-through system with a filter membrane at the bottom) demonstrated superior performance in terms of solidification and structural integrity. As shown in Figure 3B, 46 g of LS was demolded as an intact, coherent cylinder after curing. A dense CaCO_3_-cemented layer (approximately 1 cm thick) was formed in the upper section. The flow-through design, coupled with peristaltic pump injection (400 μL/min), facilitated the percolation of bacterial and cementation solutions and improved drainage. By sustaining continuous liquid renewal and efficient drainage, this configuration maintained favorable aerobic conditions for bacterial metabolism and promoted more homogeneous CaCO_3_ deposition, without the water stagnation inherent to static molds.

#### 3.2.2. Influence of Treatment Frequency on Solidification Homogeneity

With the total amounts of cementation solution and bacterial suspension kept constant, the number of treatment cycles influences the distribution and uniformity of CaCO_3_ precipitation by controlling the injection frequency. This effect was evaluated using samples solidified with *S. pasteurii* in the cylindrical flow-through mold. As shown in Figure 3C, samples subjected to seven treatment cycles (Figure 3C(b)) exhibited markedly better integrity than those treated with three treatment cycles (Figure 3C(a)). Under the seven treatment cycles, no obvious delamination was observed, and a cementation layer with a thickness of 1.5–2.0 cm was formed in the upper section. In contrast, the three treatment cycles produced a thinner cementation layer (0.8–1.0 cm) confined to the upper part, leaving loose slag in the middle and lower sections. This difference may be attributed to a repeated small-volume injection strategy, which prevented rapid pore sealing in the upper layer that typically occurred after a single large-volume injection [34,35]. A single large volume of cementation solution can quickly fill the upper interparticle pores with CaCO_3_, hindering the downward penetration of subsequent liquid and resulting in insufficient reactions in the middle and lower layers. In contrast, multiple injections at 24 h intervals generate a moderate amount of CaCO_3_ each time, ensuring effective cementation while preserving pathways for further liquid penetration.

#### 3.2.3. Solidification Capability of Different Bacterial Strains

The difference in solidification capability between the two urease-producing strains was reflected in the hardness and integrity of the macroscopic products. Under identical conditions (flow-through system, 1 M cementation solution, seven treatment cycles), samples solidified by *S. pasteurii* (Figure 3D(a)) exhibited better specimen integrity and greater resistance to crumbling, whereas those solidified by WD018 (Figure 3D(b)) were softer and more easily crumbled. These qualitative differences in macroscopic integrity are consistent with the greater extent of calcite cementation revealed by SEM and XRD (Section 3.3 and Section 3.4). Such disparity is primarily attributable to the higher urease activity of *S. pasteurii*, whose urea hydrolysis and CO_3_^2−^ generation rates were more than 20 times higher than those of WD018. The higher urease activity enabled rapid combination with Ca^2+^ from the cementation solution and LS, generating more calcite with higher crystallinity, thereby enhancing the cementation strength between LS particles.

### 3.3. Microstructural Characteristics of the Solidified Products

**Figure 4:**
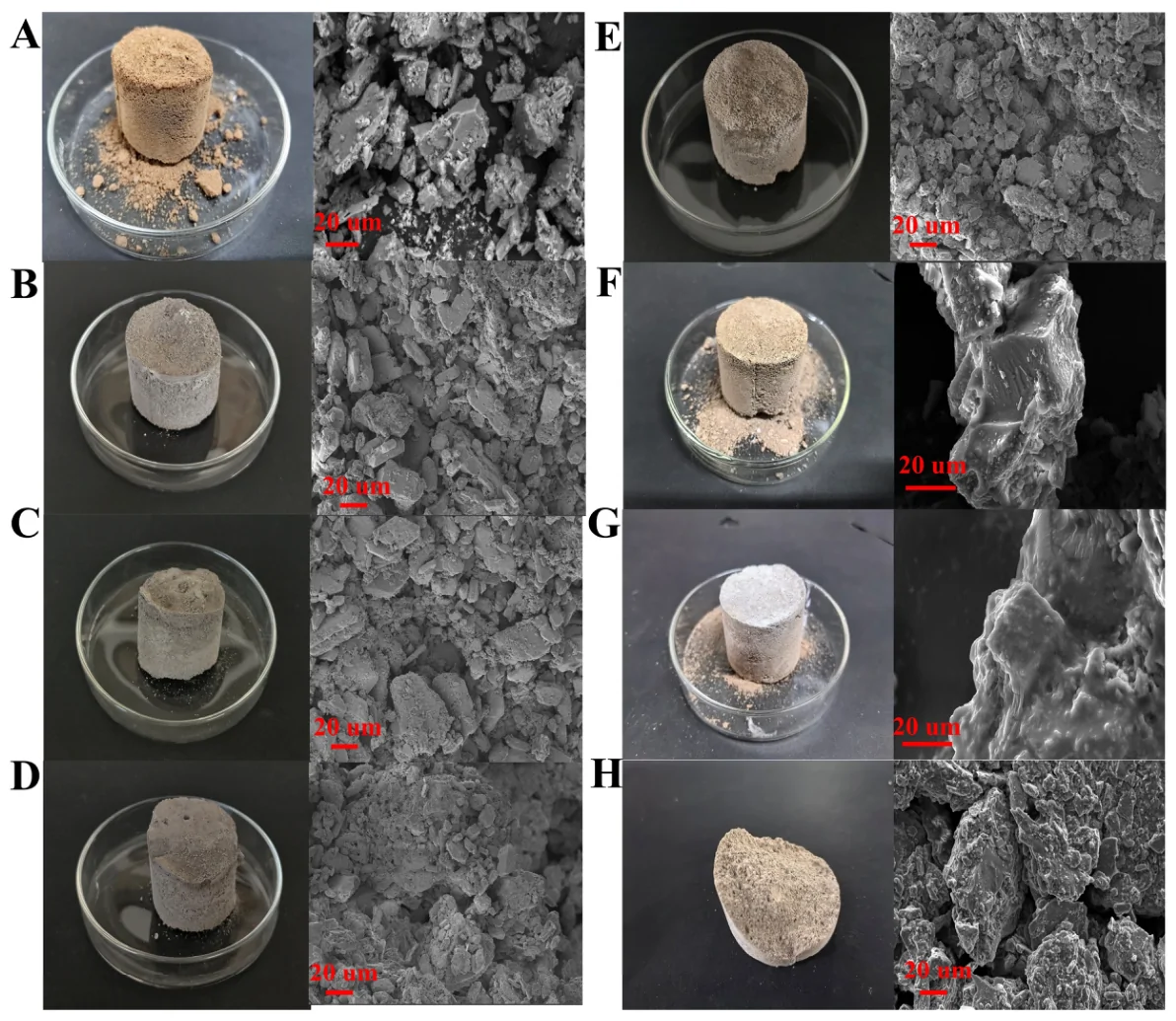
SEM morphologies of LS under different treatment conditions. (**A**) Unsolidified LS; All specimens in panels (**B**–**H**) were prepared in the cylindrical flow-through mold with 1 M cementation solution and seven treatment cycles. Blank controls: (**B**) LS treated with sterile CASO medium and cementation solution without bacteria (medium blank control); (**C**) LS treated with heat-inactivated *S. pasteurii* and cementation solution (killed-cell control); (**D**) LS treated with sterile LB medium and cementation solution without bacteria (medium blank control); (**E**) LS treated with heat-inactivated WD018 and cementation solution (killed-cell control). Live-cell treatments: (**F**) LS solidified by WD018; (**G**) LS solidified by *S. pasteurii*; (**H**) cross-section of the LS product solidified by *S. pasteurii*.

The micromorphological changes in LS before and after solidification were clearly revealed by SEM, illustrating the location, morphology, and pore-filling efficiency of the precipitated CaCO_3_. Under identical conditions (cylindrical PVC mold, 1 M cementation solution, seven treatment cycles), as shown in Figure 4A, untreated LS particles possessed rough surfaces with numerous irregular pores (mostly 5–20 μm in diameter) and no significant adherent mineral precipitation, explaining their initially high water permeability at the microscale. To establish whether such precipitation required viable bacteria, four blank controls, including two sterile-medium blanks and two heat-inactivated-cell controls (Figure 4B–E), were examined by SEM under identical conditions. In all four controls, the slag particles retained rough surfaces and highly open, interconnected pores closely resembling those of the untreated LS (Figure 4A), and no densely packed rhombic or spherical crystals were observed on particle surfaces or within pores. These SEM observations indicated that neither the culture medium, the cementation solution, nor heat-killed cells alone produced appreciable CaCO_3_ precipitation.

In clear contrast, after solidification with live WD018 (Figure 4F), numerous irregular spherical precipitates (1–3 μm in diameter) appeared on the particle surfaces, partially filling the pores, although the precipitate distribution was uneven and some small pores remained unfilled. The micromorphology of LS solidified by live *S. pasteurii* was markedly different (Figure 4G). The particle surfaces were covered with abundant angular, square, or rhombic crystalline deposits (2–5 μm in edge length), which were closely packed around the LS particles. The original irregular pores were substantially filled, with only a limited number of small residual pores remaining. The SEM images therefore revealed a greater abundance and more extensive pore filling of crystalline deposits in the *S. pasteurii*-treated samples than in the WD018-treated samples. The phase identity of these deposits was further examined by XRD analysis. The better specimen integrity observed for *S. pasteurii*-treated samples is consistent with the greater extent of pore filling observed by SEM. The cross-sectional SEM image (Figure 4H, corresponding to the *S. pasteurii*-treated samples) further showed that crystalline precipitates were distributed not only near the outer surface but also to a depth of 1.0–1.5 cm within the specimen. Pores between internal particles were also partially filled, resulting in a more continuous mineralized structure. This distribution was consistent with the more uniform mineralization observed in the flow-through treatment. Taken together, the contrast between the loose, crystal-free blanks (Figure 4B–E) and the densely cemented live-cell treatments (Figure 4F,G) supports a role for viable urease-producing bacteria in the precipitation and pore filling associated with LS solidification.

### 3.4. Phase Composition and Crystal Structure Evolution

#### 3.4.1. Phase Composition Changes Before and After Solidification

**Figure 5:**
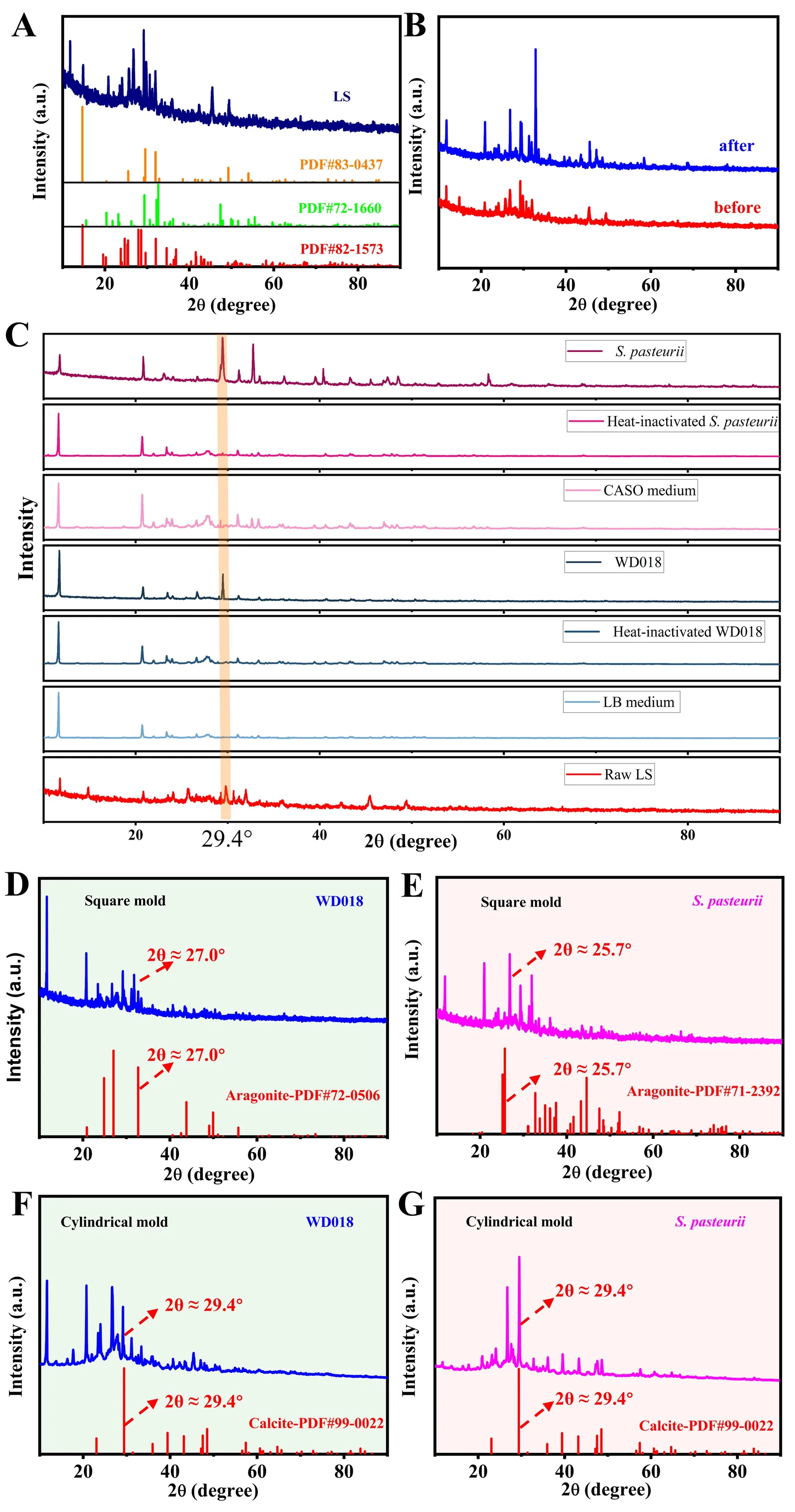
XRD patterns of LS under different treatment conditions. (**A**) Unsolidified LS; (**B**) Comparison of LS before and after solidification by WD018; (**C**) XRD patterns of four blank control samples: CASO medium plus cementation solution; heat-inactivated *S. pasteurii* plus cementation solution; LB medium plus cementation solution; heat-inactivated WD018 plus cementation solution. Note the absence of calcite reflections at 2θ ≈ 29.4° and 39.4°; (**D**) Solidified product by WD018 in the square mold; (**E**) Solidified product by *S. pasteurii* in the square mold; (F) Solidified product by WD018 in the cylindrical mold; (**G**) Solidified product by *S. pasteurii* in the cylindrical mold.

The phase composition of LS before and after solidification was characterized by XRD to qualitatively assess changes in mineral phases after MICP treatment. The XRD pattern of the raw LS (Figure 5A) confirmed quartz (SiO_2_, PDF#82-1573 [36]), calcium sulfate (CaSO_4_, PDF#83-0437 [37]), and dicalcium silicate (Ca_2_SiO_4_, PDF#72-1660 [38]) as the primary crystalline phases. No characteristic peaks corresponding to carbonate minerals were detected. Marked changes in the phase composition occurred after solidification (Figure 5B). Compared to the untreated slag, the characteristic peak intensities of quartz, gypsum, and dicalcium silicate were greatly reduced or disappeared, while distinct calcite peaks (PDF#99-0022) emerged at 2θ ≈ 29.4°, 39.4°, and 43.2° [39], consistent with urease-producing bacteria inducing CaCO_3_ formation via the MICP process. The mechanism involves CO_3_^2−^ generated by bacterial urea hydrolysis reacting not only with Ca^2+^ from the cementation solution but also with Ca^2+^ released from gypsum (CaSO_4_) and dicalcium silicate (Ca_2_SiO_4_) within the LS [40,41]. Such conversion transformed the less stable calcium-bearing phases in the slag into more stable CaCO_3_, which may contribute to the stabilization of soluble components such as sulfates.

To rule out the possibility that the calcite peaks arose from non-biological precipitation, the four corresponding blank control samples were also examined by XRD (Figure 5C). All four control patterns were essentially identical to that of the raw LS (Figure 5A), with quartz, gypsum, and dicalcium silicate remaining the dominant phases and with no detectable calcite reflections at 2θ ≈ 29.4° or 39.4°. This absence of calcite was fully consistent with the SEM findings for the same controls (Figure 4B–E), in which the slag matrix remained loose and uncemented. Together, the SEM and XRD blank-control results demonstrate that the calcite formation reported here was specifically induced by the metabolic activity of viable bacteria and could not be reproduced by the culture medium, cementation solution, or heat-inactivated cells alone.

#### 3.4.2. Crystallinity of CaCO_3_ as Influenced by Mold Type and Bacterial Strain

Mold flowability and bacterial strain type jointly determined the crystalline composition of CaCO_3_ in the solidified products, thereby influencing the stability of the solidification effect. As shown in Figure 5D,E, in the square non-flow-through mold, the XRD pattern of the WD018-solidified product showed a vaterite characteristic peak (PDF#72-0506) at 2θ ≈ 27.0°, with a weak calcite peak intensity [42,43]. Conversely, the *S. pasteurii* product exhibited calcite peaks along with an aragonite peak (PDF#71-2392) at 2θ ≈ 25.7° [44]. Since both vaterite and aragonite are metastable CaCO_3_ polymorphs that can transform into calcite under humid conditions, their presence may reduce the long-term stability of products formed in non-flow-through molds. The cylindrical flow-through mold substantially improved the CaCO_3_ crystallinity (Figure 5F,G). The vaterite peak disappeared in the WD018 product, leaving only calcite peaks. The calcite peak intensity in the *S. pasteurii* product further increased with no aragonite detected, indicating the flow-through system favors the formation of stable calcite. Furthermore, a comparison between the XRD patterns in Figure 5F,G revealed that the calcite peaks were sharper for the product solidified by *S. pasteurii*, suggesting higher CaCO_3_ crystallinity under identical mold conditions, a difference likely attributable to its higher urease activity.

The distinct crystallization pathways underscore a critical interplay between environmental conditions and biological catalysis. The flow-through design, by ensuring adequate oxygen supply and preventing metabolic by-product accumulation, likely maintained a solution chemistry conducive to direct calcite nucleation over metastable phases [45]. The synergy between engineered oxygen transfer and intrinsic bacterial activity is critical for forming a durable and stable solidification matrix, which supports its potential performance in engineering applications.

### 3.5. Effects of Cementation Solution Concentration and Treatment Cycles on Product Phase

**Figure 6:**
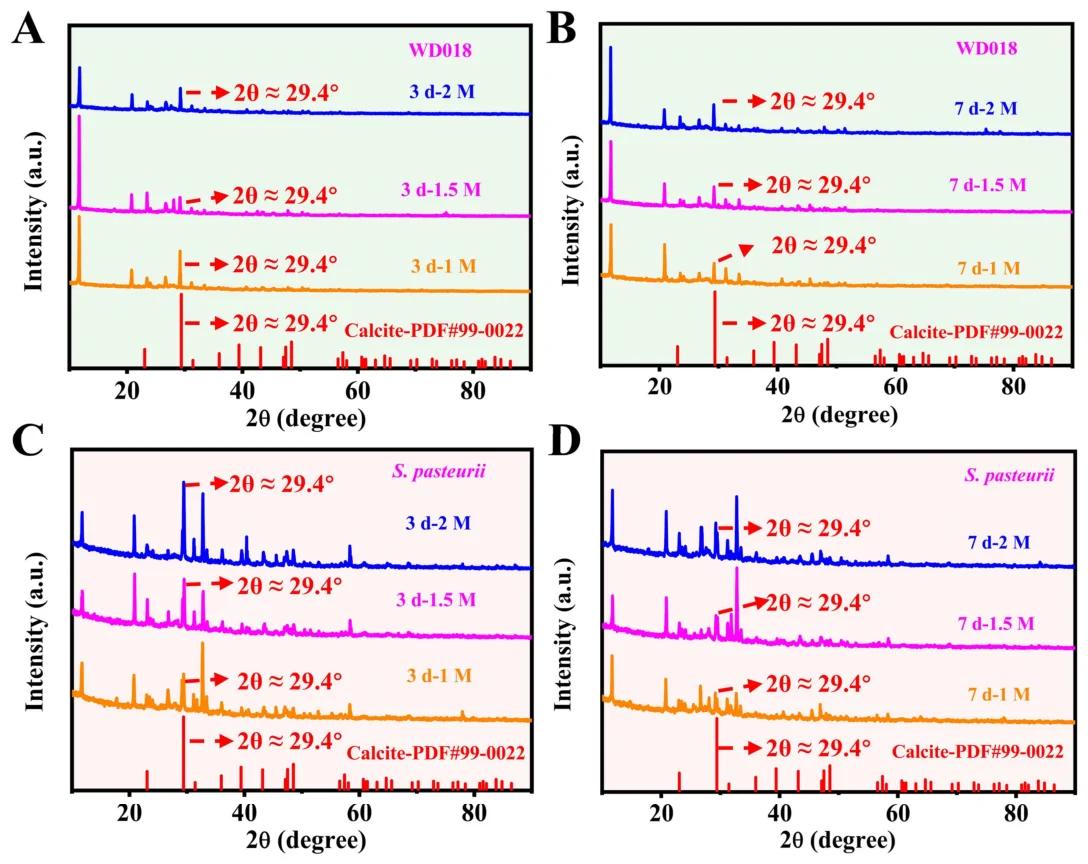
XRD patterns of solidified products under different cementation solution concentrations and treatment cycles. (**A**) WD018, three treatment cycles (3 d); (**B**) WD018, seven treatment cycles (7 d); (**C**) *S. pasteurii*, three treatment cycles (3 d); (**D**) *S. pasteurii*, seven treatment cycles (7 d).

In the WD018 system (Figure 6A,B), increasing the cementation solution concentration from 1 M to 2 M progressively enhanced the intensity of the characteristic calcite peak (2θ ≈ 29.4°). Samples subjected to seven treatment cycles at 24 h intervals exhibited higher calcite peak intensities than those treated for only three treatment cycles, indicating that repeated supplementation of the cementation solution promoted progressive calcite precipitation by providing additional Ca^2+^ and urea substrates. However, increasing the cementation solution concentration also accelerated surface clogging: at 2 M, CaCO_3_ precipitated predominantly near the upper surface, rapidly sealing the interparticle pores in the top layer, lowering the surface permeability, and impeding the downward infiltration of subsequently injected suspensions. This concentration-induced clogging offset the benefit of the additional substrates in the larger flow-through specimens. Therefore, although 2 M produced stronger calcite peaks in the small-scale square-mold tests, 1 M was selected for the larger flow-through treatment as a practical balance between carbonate precipitation and solution permeability [46,47].

The solidification system using *S. pasteurii* showed a similar trend (Figure 6C,D). The 2 M group exhibited the highest calcite peak intensity, while the group under seven treatment cycles showed more symmetrical peak shapes without interference from impurity peaks. Under the same concentration and cycle conditions, the samples solidified by *S. pasteurii* displayed stronger calcite peak intensities than those treated with WD018, which was consistent with greater calcite formation and changes in the relative prominence of the original mineral phases in the XRD patterns. Collectively, these findings underscore the distinct advantage of *S. pasteurii* in achieving effective LS solidification.

### 3.6. Physicochemical Properties of Solidified Products

#### 3.6.1. CaCO_3_ Content Distribution

The spatial distribution of CaCO_3_ content serves as an indicator of bacterial metabolic activity and oxygen accessibility within the solidified matrix. The CaCO_3_ contents in different sections of the solidified samples are summarized in Table 1.

Several interrelated factors can be invoked to explain this distribution pattern [46,48]. Firstly, oxygen availability was highest in the upper layers because of direct contact with air, favoring the aerobic metabolism of both strains and leading to preferential CaCO_3_ precipitation in this zone. Secondly, the inherently higher urease activity of *S. pasteurii* enabled it to generate more CaCO_3_ than WD018 under identical conditions, consistent with its superior macroscopic solidification performance described above.

#### 3.6.2. Water Seepage and Permeability Reduction

The hydraulic permeability of LS was evaluated to assess the effect of MICP treatment on water transport through the solidified matrix. The permeability coefficient of untreated LS ranged from (1.71 × 10^−6^) to (1.13 × 10^−5^) m/s. After MICP treatment, no visible water seepage was observed from the bottom of the specimens during the 30 min constant-head test, suggesting reduced water seepage and permeability compared with untreated LS. Accordingly, the reduction in permeability of the treated specimens was assessed from the visible water seepage during the constant-head test.

The reduction in hydraulic permeability is consistent with the microstructural characteristics of the MICP-treated LS. SEM and XRD analyses showed that CaCO_3_, predominantly in the calcite form, precipitated on LS particle surfaces and within interparticle voids. These precipitates can partially fill pore spaces and reduce the connectivity of interconnected flow pathways, thereby restricting water transport through the LS matrix. This interpretation is consistent with the observed increase in CaCO_3_ content and the formation of mineral precipitates within the treated specimens.

### 3.7. Mechanism of MICP-Based LS Solidification

The underlying mechanism for MICP-based LS solidification involves the following key steps. Urease secreted by the bacteria catalyzes the stepwise hydrolysis of urea, producing NH_3_ and H_2_CO_3_. NH_3_ reacts with water, raising the environmental pH, while H_2_CO_3_ dissociates to produce CO_3_^2−^. Finally, CO_3_^2−^ combines with Ca^2+^ from cementation solution and with Ca^2+^ released from LS, such as from the dissolution of gypsum and dicalcium silicate, to form CaCO_3_ precipitates that solidify the LS [46]. The specific reaction process is as follows:

Urea hydrolysis:

$${\mathrm{CO}({\mathrm{NH}}_{2})}_{2}+H_{2}O\to {\mathrm{NH}}_{3}+{\mathrm{NH}}_{2}\mathrm{COOH}$$

Carbamic acid hydrolysis:

NH_2_COOH + H_2_O → NH_3_ + H_2_CO_3_

Carbonic acid dissociation:

$$H_{2}{\mathrm{CO}}_{3}\to 2H^{+}+{\mathrm{CO}}_{3}^{2-}$$

Ammonia hydration:

$${\mathrm{NH}}_{3}{+H}_{2}O⇌{\mathrm{NH}}_{4}^{+}+{\mathrm{OH}}^{-}$$

Overall precipitation reaction:

$${\mathrm{Ca}}^{2+}+{\mathrm{CO}}_{3}^{2-}={\mathrm{CaCO}}_{3}↓$$

In the aqueous phase, the ammonia generated during urea hydrolysis is rapidly protonated to form ammonium ions (NH_4_^+^), which constitute the principal nitrogen-containing by-product of ureolysis-driven MICP. This ammonium remains in the effluent and therefore requires appropriate management or recovery in field-scale applications.

The key difference in the solidification efficacy between the two strains lies in their urease activity. The high urease activity of *S. pasteurii* accelerates urea hydrolysis, increasing the rate of CO_3_^2−^ generation and thereby promoting the crystallization of calcite. In contrast, the lower urease activity of WD018 results in the predominant formation of metastable vaterite. Among environmental factors, oxygen availability may be an important factor regulating bacterial activity. The flow-through mold design may have alleviated local oxygen limitation and facilitated bacterial metabolic activity. Furthermore, the decrease in silicate- and sulfate-related signals suggests that MICP altered the relative abundance of the original mineral phases, which is expected to favor the stabilization of the soluble components within the slag matrix.

## 4. Conclusions

The solidification of LS was achieved using MICP technology, demonstrating the feasibility of microbial-induced mineralization for LS stabilization under the tested conditions. Performance comparisons between the laboratory-isolated strain, *Lysinibacillus* sp. WD018, and the model ureolytic bacterium *S. pasteurii* revealed that bacterial urease activity was associated with differences in carbonate precipitation. The substantially higher urease activity of *S. pasteurii* (approximately 20-fold greater than WD018) was associated with greater carbonate precipitation, higher CaCO_3_ production, and stronger calcite signals in the XRD patterns. Process parameters critically influenced the distribution and extent of mineral precipitation during LS treatment. A flow-through cylindrical mold, combined with a 1 M cementation solution and repeated treatment at 24 h intervals, provided favorable conditions for bacterial mineralization and promoted more uniform carbonate deposition within the slag matrix under the tested conditions. Overall, the results demonstrated that the markedly higher urease activity of *S. pasteurii* was associated with enhanced carbonate precipitation and stronger calcite formation, while the flow-through configuration and repeated treatment favored a more uniform distribution of the precipitated minerals. These results provide a useful basis for further investigation of microbial mineralization processes in LS stabilization. To advance the engineering application of MICP-based LS solidification, further work should evaluate the mechanical properties, leaching behavior, and environmental stabilization of treated LS, together with long-term durability and ammonium by-product management, and should demonstrate the feasibility of scale-up.

## Figures and Tables

**Table 1:** CaCO_3_ contents in solidified LS samples. Values are mean ± standard deviation (*n* = 3).

Strain	Temperature (°C)	Upper Section (%)	Middle Section (%)	Lower Section (%)
*S. pasteurii*	30	51 ± 0.91	23 ± 0.23	23 ± 0.75
WD018	30	32 ± 0.10	15 ± 0.35	15 ± 0.47

## Data Availability

The data presented in this study are available on request from the corresponding author due to restrictions.
